# First Evaluation of Insecticide Efficacy Against the Invasive Two-Spot Cotton Leafhopper (*Amrasca biguttula* [Hemiptera: Cicadellidae]) on Ornamental Hibiscus in the United States

**DOI:** 10.3390/insects17040358

**Published:** 2026-03-25

**Authors:** Nisha Yadav, Peilin Tan, Muhammad Z. Ahmed

**Affiliations:** Pee Dee Research and Education Center, Clemson University, Florence, SC 29506, USA

**Keywords:** hopperburn, sap-feeding pest, nursery production, residual toxicity, sequential-cohort bioassay, systemic insecticides, ornamental entomology, invasive species management, hibiscus host suitability, regulatory plant movement restrictions

## Abstract

The two-spot cotton leafhopper (TSCL), *Amrasca biguttula* (Hemiptera: Cicadellidae), is an emerging invasive pest in the southeastern United States. Although TSCL has historically been associated with cotton and vegetable crops, recent detections on ornamental hibiscus have raised regulatory concern, including “Stop Sale and Hold” orders and an emergency quarantine in Texas. Despite increasing pressure on hibiscus, no insecticide efficacy data exist for ornamental systems. We evaluated the acute (0–24 h) and residual (24–96 h) toxicity of bifenthrin, flupyradifurone, and tolfenpyrad against adult and immature TSCL using a sequential-cohort leaf-disc bioassay. New insects were introduced at 24 h and 72 h to isolate residue-based mortality from prolonged exposure effects. Bifenthrin caused the highest acute mortality at 24 h, whereas flupyradifurone and tolfenpyrad exhibited slower initial activity but strong residual performance. Immatures were more susceptible than adults across all doses. By 72 h, all three insecticides produced near-complete mortality, with significant treatment and dose effects confirmed by ANOVA and binomial GLM analyses. Dose–response curves showed steep concentration-dependent mortality for bifenthrin and tolfenpyrad and a time-dependent response for flupyradifurone. These results provide the first insecticide efficacy data for TSCL on ornamental hibiscus and offer immediate guidance for nursery producers and regulatory agencies. The findings establish a foundation for whole-plant and greenhouse evaluations to support integrated management and interstate plant-movement compliance.

## Simple Summary

The two-spot cotton leafhopper (TSCL) is a newly emerging invasive pest now affecting ornamental hibiscus in the southeastern United States. Its recent spread has raised grower and regulatory concern, including plant-movement restrictions and quarantine actions. Because no insecticide information was available for ornamental systems, we tested three commonly used products to measure how quickly they kill TSCL and how long their residues remain effective. Bifenthrin caused the fastest mortality, while flupyradifurone and tolfenpyrad provided strong residual control over several days. Young stages were more susceptible than adults. These results provide the first guidance for managing TSCL on ornamental hibiscus and support growers and regulators facing this rapidly emerging pest.

## 1. Introduction

*Amrasca biguttula* (Ishida) (Hemiptera: Cicadellidae), hereafter referred to as the two-spot cotton leafhopper (TSCL), is an emerging invasive pest of increasing concern in the southeastern United States. Native to South and Southeast Asia, TSCL is a destructive pest of cotton, okra, eggplant, potato, roselle, and other crops, where feeding by nymphs and adults causes hopperburn, leaf necrosis, stunting, and substantial yield loss [1,2]. In its native and expanded ranges, TSCL has also developed resistance to multiple insecticide classes, including pyrethroids, organophosphates, and neonicotinoids, complicating management efforts [3,4,5].

TSCL was first detected in the Western Hemisphere in Puerto Rico in 2023 [6] and subsequently in Florida in 2024 [7]. By mid-2025, coordinated surveys documented TSCL in more than 100 counties across Alabama, Florida, Georgia, and South Carolina, with injury ranging from mild chlorosis to severe hopperburn and defoliation [8]. Recent phylogeographic analysis revealed that U.S. populations represent a single mtCOI haplotype identical to Hap01, the globally dominant lineage, indicating a recent introduction and rapid regional spread [9]. The pest has now been confirmed on cotton, okra, eggplant, hibiscus, and several weedy hosts, raising concerns about establishment, overwintering, and spillover into high-value crops [10].

Regulatory agencies have responded aggressively to TSCL detections. In 2025, the Texas Department of Agriculture enacted an emergency quarantine to prevent the movement of TSCL-infested plant material after detections in nursery and landscape settings raised concerns about rapid spread and potential economic losses to cotton, vegetables, and ornamentals. The quarantine restricted the movement of host plants, required mandatory inspections, and emphasized that TSCL poses a significant risk to interstate commerce and agricultural production. These actions highlight the vulnerability of ornamental pathways—particularly hibiscus—to long-distance movement of TSCL and underscore the need for validated management tools suitable for nursery systems.

Although TSCL is historically recognized as a cotton and vegetable pest, recent reports indicate that ornamental hibiscus (*Hibiscus rosa-sinensis*) has become a key host in the southeastern U.S. Commercial nurseries have reported rapid hopperburn development, plant decline, and regulatory concern due to “Stop Sale and Hold” orders triggered by TSCL detections [11]. Hibiscus is widely marketed as a pollinator-friendly, low-input ornamental, and many nurseries avoid insecticide applications on flowering plants to maintain pollinator-safe labeling [12]. This creates a management gap: hibiscus plants can harbor undetected TSCL populations, serve as overwintering refugia, and act as bridge hosts enabling early-season infestations in nearby cotton and vegetable fields. Because hibiscus is shipped across state lines year-round, infested plants also pose a regulatory risk for interstate movement.

Despite the increasing importance of hibiscus as a reservoir host, nearly all published insecticide efficacy studies for TSCL have been conducted on cotton, okra, eggplant, or other vegetable crops. No standardized chemical efficacy data exist for ornamental hibiscus, even though nursery production systems differ markedly from field crops in canopy structure, irrigation practices, residue persistence, and application constraints. This lack of information limits the ability of growers, regulators, and extension specialists to implement effective management programs for ornamental systems and to prevent the movement of infested plants through interstate commerce.

To address this gap, we selected three insecticides—bifenthrin (IRAC 3A), flupyradifurone (IRAC 4D), and tolfenpyrad (IRAC 21A)—because they represent major modes of action currently available, labeled for use, and realistically applicable in U.S. nursery and landscape systems for piercing–sucking pests. These products differ in speed of kill, systemic activity, and residual persistence, providing growers with rotation options essential for resistance management. They also reflect the chemistries most frequently used or requested by hibiscus producers facing TSCL outbreaks [11].

We conducted a leaf-disc laboratory bioassay because it provides a standardized, containment-safe method for evaluating acute and residual toxicity while eliminating confounding nursery variables such as irrigation leaching, uneven spray coverage, and canopy architecture. Leaf-disc assays allow precise control of dose, exposure, and timing, enabling clear separation of acute toxicity (0–24 h) from residual activity (24–72 h and 72–96 h). This approach is widely used for sap-feeding pest toxicology and is the required first step before greenhouse or nursery-scale trials.

Given the rapid spread of TSCL in the southeastern U. S., the increasing frequency of hibiscus infestations, the regulatory actions triggered by its detection, and the absence of insecticide performance data for ornamental hosts, this study provides the first evidence-based evaluation of insecticide efficacy against TSCL on hibiscus. Our results offer immediate guidance for nursery producers and regulatory agencies and establish a foundation for future whole-plant and field-scale evaluations.

## 2. Materials and Methods

### 2.1. Insects

Adults and immatures of the two-spot cotton leafhopper (TSCL) were originally collected from field-grown cotton at the Clemson University Pee Dee Research and Education Center in Florence, South Carolina. A supplemental colony was then maintained on unsprayed hibiscus plants (*Hibiscus rosa sinensis* ‘Hot Shot’) at the same location. All insects used in the bioassays were taken from these hibiscus plants. Hibiscus plants of uniform size and age were obtained from a commercial nursery on 22 August 2025 and transplanted into 5-gallon pots containing a commercial substrate. Insects were collected with a mouth aspirator to avoid mechanical injury. They were immediately transferred into ventilated plastic containers filled with untreated hibiscus leaves. All insects were held for one hour at 26 ± 1 °C, 70–80% RH, and a 14:10 (L:D) photoperiod to recover from handling stress. Only active and undamaged individuals were used. Adults and immatures were tested in separate experiments, but all procedures and environmental conditions were identical.

### 2.2. Insecticides and Dose Preparation

Three insecticides were selected to represent the major IRAC groups and different modes of action relevant to TSCL management. Tolfenpyrad (Hachi Hachi SC, IRAC Group 21A) is a mitochondrial complex I electron transport inhibitor. It acts mainly by contact and shows translaminar movement in treated foliage. Flupyradifurone (Altus, IRAC Group 4D) is a butenolide nicotinic acetylcholine receptor modulator. It is systemic, xylem-mobile, and also moves translaminarly. Bifenthrin (Talstar Professional, IRAC Group 3A) is a pyrethroid that modulates sodium channels. It acts by contact and forms persistent surface residues but has no systemic or translaminar activity.

Five dose levels were prepared for each insecticide. These doses were calculated from the median label rate used in our field experiment [13]. The same median label rates were also used in our previously published field study on TSCL management (Yadav et al. 2026) [13], ensuring consistency between laboratory and field evaluations. For each product, the median value within the EPA-registered application range was converted to mL L^−1^, and the five-level dose series (D1–D5) was generated by scaling this field-relevant rate proportionally. This approach ensures that all concentrations tested reflect realistic application intensities rather than artificially elevated laboratory doses. Tolfenpyrad was tested at 0.80, 1.26, 2.00, 3.16, and 5.00 mL L^−1^ (T1D1–T1D5). Flupyradifurone was tested at 0.12, 0.21, 0.35, 0.59, and 1.00 mL L^−1^ (T2D1–T2D5). Bifenthrin was tested at 0.20, 0.30, 0.45, 0.67, and 1.00 g L^−1^ (T3D1–T3D5). An untreated control was included at each dose position (T4D1–T4D5). All solutions were prepared fresh with distilled water.

### 2.3. Leaf Disc Treatment

Hibiscus leaves of uniform age and size were selected at the newly fully expanded stage. Leaves were rinsed with distilled water and air-dried at room temperature until all surface moisture had evaporated. Whole leaves were then dipped into the treatment solution until fully immersed and removed. Treated leaves were placed directly into 9 cm plastic Petri dishes. Each Petri dish lid had a 3 cm-diameter hole covered with a no-thrips screen to allow ventilation (Figure S1). Leaves were allowed to air-dry at room temperature for 45 min following insecticide application. Adult and immature TSCL were collected using a standard mouth aspirator into 4–5 mL polypropylene collection vials (≈12–16 mm diameter). To suppress activity and prevent escape during transfer, the vials were placed on crushed ice for approximately five minutes, inducing transient cold immobilization. After drying, live adults and immatures were transferred to control and treated leaves using a fine camel-hair brush, with careful handling to minimize stress and reduce natural mortality.

### 2.4. Experimental Design

The experiment followed a randomized complete block design with five blocks. Within each block, all insecticide × dose combinations and the corresponding control positions (4 treatments × 5 dose levels = 20 combinations) were represented once and assigned to Petri dishes (DishID) in random order. Each insecticide × dose combination was replicated three times across blocks, and DishID served as the experimental unit. For each DishID and each cohort, five TSCL were introduced into each Petri dish. Adults and immatures were tested in separate experiments using identical randomization and blocking. A randomized complete block design was selected instead of a completely randomized design because preliminary observations indicated block-level variation in insect vigor, leaf condition, and microenvironmental factors. Blocking allowed this structured variability to be accounted for explicitly, reducing residual error and increasing the precision of treatment comparisons relative to a CRD.

### 2.5. Acute Toxicity Assay (0–24 h)

Acute toxicity was evaluated using a first cohort of TSCL exposed immediately to freshly treated leaf discs. Five insects were introduced into each Petri dish and placed onto the treated leaf disc using a mouth aspirator and a fine camel-hair brush. Petri dishes were sealed with Parafilm^®^ and maintained at 26 ± 1 °C, 70–80% RH, and a 14:10 L:D photoperiod. Mortality for this first cohort was recorded at 5 h and 24 h after introduction. Insects were considered dead if they failed to move when gently probed with a fine brush. Immediately after the 24 h assessment, all insects from the first cohort were removed and discarded, and the treated discs remained in the dishes for the next cohort.

### 2.6. Residual Toxicity Assay (24–72 h and 72–96 h)

Residual toxicity was evaluated using two sequential cohorts of TSCL, each representing a distinct exposure group introduced at a different residue age rather than repeated observations on the same insects. The second cohort assessed the toxicity of 24 h-old residues. This cohort was independent of the first cohort and was used to isolate mortality caused by aged residues rather than prolonged exposure. At 24 h after treatment, five new insects were introduced into each Petri dish onto the same treated leaf discs used in the acute assay. Dishes were resealed with Parafilm^®^ and maintained under the same environmental conditions. Mortality of this second cohort was recorded at 72 h after treatment, corresponding to approximately 48 h of exposure to 24 h-old residues. Thus, the 72 h time point reflects mortality of a new cohort exposed to 24 h-old residues, not continued survival of the first cohort. Immediately after the 72 h assessment, all second-cohort insects were removed and discarded, and the treated discs remained in the dishes.

A third cohort was used to evaluate the toxicity of older residues, again representing a new group of insects introduced specifically to measure mortality on 72 h-old residues. At 72 h after treatment, five new insects were introduced into each Petri dish onto the same treated discs. Dishes were resealed and maintained under the same environmental conditions. Mortality of this third cohort was recorded at 96 h after treatment, corresponding to approximately 24 h of exposure to 72 h-old residues. The 48 h exposure window used for the second cohort was selected to capture potential delayed mortality on relatively fresh residues, whereas the shorter 24 h window for the third cohort provided a conservative test of lethality on older residues without confounding residue decay with prolonged exposure. Accordingly, the 96 h time point reflects mortality of this third cohort and should be interpreted as residue-age-specific mortality rather than a continuation of earlier observations. Adults and immatures were analyzed independently because they differ in cuticle thickness, feeding intensity, body size, and detoxification capacity, which produce distinct susceptibility profiles despite feeding on the same leaf surface.

### 2.7. Data Processing and Statistical Analysis

All analyses were conducted in R (R Core Team, Vienna, Austria) [14]. For each DishID and time point, the number of dead and alive TSCL individuals (out of five) was recorded. Acute (5 and 24 h) and residual (72 and 96 h) assays were analyzed separately because they represent distinct exposure cohorts. Mortality was corrected for control mortality using Abbott’s formula [15].

Because mortality in most insecticide treatments reached 100% by 24 h and remained uniformly high across doses and time points, the data did not exhibit the graded responses required for probit or log-logistic dose–response modeling. Consequently, LC_50_ estimation was not appropriate for this dataset and was not attempted. Instead, analyses focused on models and summary metrics suitable for datasets with complete or near-complete mortality.

Corrected mortality at each time point was analyzed using generalized linear models (GLMs) with a binomial error distribution and logit link. Insecticide, dose, and their interaction were treated as fixed effects. Overdispersion was assessed by comparing residual deviance with residual degrees of freedom; when present, an observation-level random effect was added to account for extra-binomial variation. For each time point, treatment effects were further evaluated using ANOVA, and pairwise comparisons were adjusted using Tukey’s method (α = 0.05). To summarize insecticide performance under conditions of uniformly high mortality, two descriptive potency metrics were calculated: (1) Minimum Effective Dose (MED), defined as the lowest tested dose achieving ≥ 90% mean mortality, and (2) T90, the earliest time point at which ≥90% mean mortality was observed for each insecticide × life stage combination.

Survival across the full assay period was analyzed using Kaplan–Meier estimators.

Individual-level survival times were reconstructed from cumulative mortality counts, and survival curves were compared descriptively across insecticides and life stages. All graphical outputs included corrected mortality trajectories, dose-indexed mortality patterns at 24 h, and Kaplan–Meier survival curves. Model diagnostics (residual plots, dispersion checks, and fit statistics) were examined to verify appropriate model performance.

## 3. Results

### 3.1. Acute Toxicity (0–24 h)

Mortality of immature and adult TSCL increased rapidly during the first 24 h after exposure to freshly treated hibiscus leaf discs. At 5 h, insecticide treatments differed significantly for immatures (F_3_,_40_ = 19.35; *p* = 0.45).

By 24 h, treatment effects were highly significant for immatures (F_3_,_40_ = 196.0; *p* = 0.18). Mortality patterns over time for adults and immatures are shown in Figure S2. Raw mortality counts for all replicates are provided in Supplementary Table S1.

Dose–response analysis was conducted only for the 24 h time point because it was the only period with sufficient dose-dependent variation. Mortality at 5 h was too low for model fitting, and mortality at 72 and 96 h reached 100% for all insecticides, eliminating dose separation. The 24 h dose–response curves for adults and immatures are presented in Figures S3 and S4.

### 3.2. Residual Toxicity (24–72 h and 72–96 h)

Residual toxicity increased sharply after 24 h. By 72 h, mortality reached 100% for all insecticides and both life stages. This resulted in artificially inflated F-values due to zero residual variance for adults (F_3,40_ = 2.66 × 10^31^; *p* < 0.001) and immatures (F_3,40_ = 2.66 × 10^31^; *p* < 0.001) (Table 1 and Table 2). Dose effects at 72 h were statistically significant (*p* ≈ 0.0087), but these differences were biologically irrelevant because all treatments produced complete mortality. Treatment × Dose interactions were also significant at 72 h (*p* < 0.001), driven by numerical artifacts from zero residual variance.

**Figure 1:**
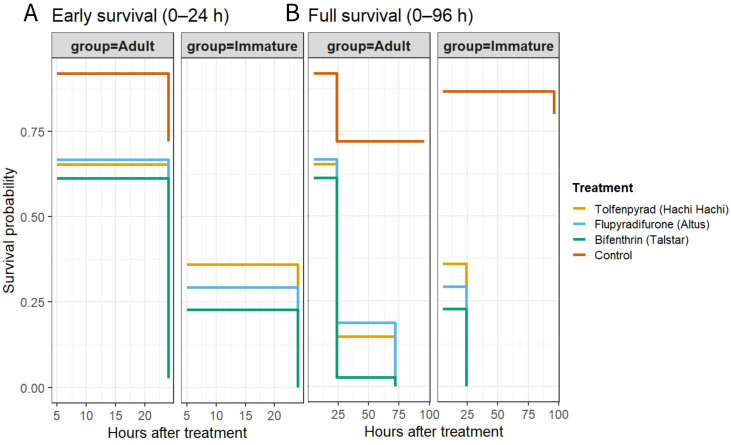
Kaplan–Meier survival curves for adult and immature *Amrasca biguttula* exposed to three insecticides and an untreated control. Individual-level survival times were reconstructed from cumulative mortality counts (five insects per dish, three dishes per treatment), and Kaplan–Meier estimators were fitted for each insecticide × life-stage combination. (**A**) highlights early survival (0–25 h), the interval where treatment-driven differences emerge most clearly. (**B**) presents the full survival curve (0–96 h), with emphasis on the later phase (25–96 h) to illustrate the extended mortality progression after the initial divergence. Step functions depict survival probabilities, with facets separating adult and immature stages and colors denoting insecticides. Immatures showed the most rapid decline in survival across treatments, while adults exhibited slower but still pronounced mortality loss. These curves complement the corrected-mortality analyses by visualizing both early-phase separation and longer-term residual effects.

At 96 h, treatment effects remained significant (*p* < 0.0001), but mortality reached 100% across all insecticides. Survival curves for both life stages under each insecticide treatment are shown in Figure 1.

### 3.3. Minimum Effective Dose (MED) and Time to 90% Mortality (T90)

Minimum effective dose (MED) and time to 90% mortality (T90) values are summarized in Table 3. Immatures reached ≥90% mortality at low doses for all insecticides. Adults required higher doses for bifenthrin but responded rapidly to tolfenpyrad and flupyradifurone. Empty cells in Table 3 indicate cases where ≥90% mortality occurred at all doses or all time points, preventing identification of a minimum effective dose or the earliest time to 90% mortality.

### 3.4. Generalized Linear Model (GLM)

The binomial GLM detected strong main effects of life stage, treatment, dose, and time (HAT), along with numerous higher-order interactions. Many coefficients were extremely large due to complete mortality at later time points, which is expected under logistic models with separation. The GLM results support the ANOVA findings and confirm that treatment and exposure time were the primary drivers of mortality.

## 4. Discussion

This study provides the first insecticide efficacy data for TSCL on ornamental hibiscus in the U.S. Although TSCL is a well-known pest of cotton and vegetables in Asia [1,2], its rapid establishment in the southeastern U.S. [6,8] and recent detections in nursery systems have created an urgent need for management recommendations specific to ornamental production. Until now, no data existed to guide chemical control on hibiscus, despite its role as a high-risk reservoir and a major pathway for interstate movement. Our results directly address this gap and provide the first evidence-based framework for managing TSCL in nursery environments.

A sequential-cohort design was essential to accurately quantify acute and residual activity. The insects evaluated at 72 h and 96 h were not the same individuals tested at 24 h. Instead, new cohorts were introduced onto the same treated leaf discs after each observation period. This design eliminates starvation-related mortality, age-related decline, and cumulative sublethal effects that would otherwise inflate mortality estimates. TSCL survival declines rapidly in the absence of feeding, and holding the same insects for 96 h would have produced mortality unrelated to chemical activity. By introducing new insects at 24 h and again at 72 h, mortality at 72 h and 96 h reflects true residue-based toxicity. This approach is widely used for sap-feeding pests because it isolates chemical persistence on the substrate from the biology of the insects themselves. It provides a more rigorous and defensible estimate of residual performance than single-cohort assays.

An alternative framework introduces insects at several residue ages and measures mortality at fixed intervals such as 6, 12, 24, 48, and 72 h. This creates a clear matrix of residue age and exposure duration. It separates chemical decay from time-dependent physiological decline. It also captures residue weathering and canopy effects that occur under greenhouse or whole-plant conditions. Our study used a sequential-cohort design because it isolates acute and residue-based mortality under controlled conditions and avoids starvation or age-related artifacts. The more granular residue-age × exposure-duration approach is a logical next step for refining residual performance under production-scale environments.

Bifenthrin produced the highest acute mortality at 24 h for both adults and immatures, consistent with the rapid neurotoxic action of pyrethroids reported in other leafhopper systems [3]. Flupyradifurone and tolfenpyrad exhibited slower initial activity, which aligns with their systemic and metabolic modes of action. Immatures were more susceptible than adults, likely due to thinner cuticles, higher feeding rates, and reduced detoxification capacity. These patterns reinforce the importance of targeting early instars whenever possible and highlight the biological relevance of life-stage-specific susceptibility.

These life-stage differences are consistent with known physiological traits of hemipterans. Immatures typically possess thinner and less sclerotized cuticles, higher relative feeding rates, and reduced constitutive detoxification capacity, all of which increase internal dose per unit exposure and contribute to greater susceptibility [16,17].

These life-stage differences are consistent with known physiological traits of hemipterans. Immatures typically possess thinner cuticles, higher relative feeding rates, and lower constitutive detoxification enzyme activity, all of which increase internal dose per unit exposure. These mechanisms provide a biological basis for the consistently higher susceptibility observed in immature TSCL.

Apparent residual mortality increased sharply after 24 h for all insecticides, with near-complete mortality by 72 h. This pattern reflects the feeding biology of TSCL and the time required for systemic and translaminar chemistries to penetrate leaf tissues. The significant treatment and dose effects at 72 h confirm that these products provide strong residual control once fully expressed. Dose–response curves at 24 h showed steep concentration-dependent mortality for bifenthrin and tolfenpyrad, and a time-dependent response for flupyradifurone. These findings align with reports from cotton and okra systems in Asia, where flupyradifurone provides strong control but requires sustained feeding exposure [5,12]. Together, these results demonstrate that hibiscus foliage does not diminish the inherent toxicity or residual expression of these chemistries.

TSCL infestations on hibiscus pose unique challenges for nursery producers. Hibiscus is widely marketed as a pollinator-friendly ornamental, and many growers avoid insecticide applications on flowering plants to maintain pollinator-safe labeling [11]. This increases reliance on systemic products applied during vegetative stages. Our results show that flupyradifurone and tolfenpyrad provide strong residual control, making them suitable for prebloom applications in nursery systems. Bifenthrin provides rapid knockdown but may be less compatible with pollinator-sensitive marketing practices. These distinctions are critical for developing rotation programs that balance efficacy, pollinator safety, and regulatory compliance.

Regulatory actions further elevate the importance of effective management. TSCL detections have triggered “Stop Sale and Hold” orders in multiple states, and Texas implemented an emergency quarantine in 2025 to prevent movement of infested plant material through the nursery trade. Because hibiscus is shipped year-round across state lines, infested plants represent a high-risk pathway for long-distance dispersal. Effective chemical suppression at the nursery level is therefore essential not only for crop protection but also for regulatory compliance and interstate commerce.

Hibiscus can support high TSCL populations and provides year-round foliage in warm regions, enabling overwintering and early-season buildup [11]. This reservoir role has been documented in Puerto Rico, Florida, and South Carolina [7,11]. Our findings confirm that TSCL readily feeds and survives on hibiscus foliage, reinforcing the need for targeted management in ornamental systems. The strong residual activity observed for all three insecticides suggests that hibiscus can be effectively protected with properly timed applications.

Leaf-disc assays allowed precise control of dose, exposure, and timing, enabling clear separation of acute and residual effects. Although whole-plant studies are needed to evaluate spray coverage, phytotoxicity, and persistence under nursery irrigation regimes, leaf-disc assays are the standard first step for sap-feeding pest toxicology and provide essential baseline data. Future work should evaluate whole-plant performance, compatibility with biological control agents, and rotation programs to delay resistance.

The high mortality observed across most doses also reflects the inherent exposure intensity of the leaf-dip method, which produces uniform residue coverage and limits behavioral avoidance. While this may exceed exposure heterogeneity under nursery conditions, the doses tested were based on median label rates used in our field experiment, and the strong mortality response is consistent with field observations [13]. Whole-plant studies will be important for refining exposure realism under production irrigation and canopy structure.

This study provides foundational data for TSCL management on ornamental hibiscus and supports the development of integrated strategies for nursery producers and regulatory agencies. It establishes a scientifically rigorous baseline for future greenhouse and field evaluations and provides the first actionable chemical control recommendations for a pest of growing regulatory and economic importance.

## 5. Conclusions

This study establishes the first baseline insecticide performance data for TSCL on ornamental hibiscus in the U.S. By using a sequential-cohort design, we generated clear estimates of acute toxicity and residue-based mortality without confounding effects from prolonged exposure. All three insecticides—bifenthrin, flupyradifurone, and tolfenpyrad—were effective on hibiscus foliage, with differences in speed of kill and residual expression that can guide product selection and application timing in nursery systems. These results provide growers and regulators with actionable information for managing TSCL on a high-risk ornamental host and support the development of integrated programs that reduce the likelihood of plant-movement restrictions. Future work should evaluate whole-plant performance, irrigation effects, and compatibility with biological control to refine management strategies for this invasive pest.

## Figures and Tables

**Table 1:** Abbott-corrected mortality of immature *Amrasca biguttula* across insecticides, doses, and time points. Values represent SD mortality (n = 3 dishes per treatment). Acute (5–24 h) and residual (72–96 h) periods represent distinct exposure cohorts, not repeated observations on the same insects. The 5 h and 24 h values correspond to the first cohort introduced immediately after treatment. The 72 h values correspond to a second cohort introduced at 24 h onto 24 h-old residues, and the 96 h values correspond to a third cohort introduced at 72 h onto 72 h-old residues. Thus, each time point reflects residue age at evaluation, rather than continuous tracking of a single cohort. The final row summarizes the overall statistical significance from GLM/ANOVA models at 5 h. Dose labels (D1–D5) correspond to the five concentrations tested for each insecticide: Tolfenpyrad = 0.80, 1.26, 2.00, 3.16, and 5.00 mL L^−1^; Flupyradifurone = 0.12, 0.21, 0.35, 0.59, and 1.00 mL L^−1^; Bifenthrin = 0.20, 0.30, 0.45, 0.67, and 1.00 g L^−1^. An untreated control was included at each dose position (T4D1–T4D5).

Treatment	Dose	5	24	72	96
Bifenthrin (Talstar)	D1	60.0 ± 20.0 a	100.0 ± 0.0 a	100.0 ± 0.0 a	100.0 ± 0.0 a
	D2	73.3 ± 11.5 a	100.0 ± 0.0 a	100.0 ± 0.0 a	100.0 ± 0.0 a
	D3	73.3 ± 23.1 a	100.0 ± 0.0 a	100.0 ± 0.0 a	100.0 ± 0.0 a
	D4	93.3 ± 11.5 a	100.0 ± 0.0 a	100.0 ± 0.0 a	100.0 ± 0.0 a
	D5	86.7 ± 23.1 a	100.0 ± 0.0 a	100.0 ± 0.0 a	100.0 ± 0.0 a
Control	D1	0.0 ± 0.0 b	0.0 ± 0.0 b	0.0 ± 0.0 b	0.0 ± 0.0 b
	D2	6.7 ± 11.5 b	0.0 ± 0.0 b	0.0 ± 0.0 b	33.3 ± 57.7 b
	D3	26.7 ± 46.2 b	0.0 ± 0.0 b	0.0 ± 0.0 b	0.0 ± 0.0 b
	D4	0.0 ± 0.0 b	0.0 ± 0.0 b	0.0 ± 0.0 b	0.0 ± 0.0 b
	D5	33.3 ± 57.7 b	33.3 ± 57.7 b	0.0 ± 0.0 b	0.0 ± 0.0 b
Flupyradifurone (Altus)	D1	60.0 ± 34.6 a	100.0 ± 0.0 a	100.0 ± 0.0 a	100.0 ± 0.0 a
	D2	40.0 ± 34.6 a	100.0 ± 0.0 a	100.0 ± 0.0 a	100.0 ± 0.0 a
	D3	66.7 ± 30.6 a	100.0 ± 0.0 a	100.0 ± 0.0 a	100.0 ± 0.0 a
	D4	93.3 ± 11.5 a	100.0 ± 0.0 a	100.0 ± 0.0 a	100.0 ± 0.0 a
	D5	93.3 ± 11.5 a	100.0 ± 0.0 a	100.0 ± 0.0 a	100.0 ± 0.0 a
Tolfenpyrad (Hachi Hachi)	D1	33.3 ± 23.1 a	100.0 ± 0.0 a	100.0 ± 0.0 a	100.0 ± 0.0 a
	D2	46.7 ± 30.6 a	100.0 ± 0.0 a	100.0 ± 0.0 a	100.0 ± 0.0 a
	D3	86.7 ± 11.5 a	100.0 ± 0.0 a	100.0 ± 0.0 a	100.0 ± 0.0 a
	D4	66.7 ± 23.1 a	100.0 ± 0.0 a	100.0 ± 0.0 a	100.0 ± 0.0 a
	D5	86.7 ± 11.5 a	100.0 ± 0.0 a	100.0 ± 0.0 a	100.0 ± 0.0 a
Statistical analysis (Treatment effect)		*F* = 19.35, *p* < 0.0001	*F* = 196.00, *p* < 0.0001	*F* = 2.66 × 10^31^, *p* < 0.0001	*F* = 196.00, *p* < 0.0001

Note: Letters indicate mean separation among insecticide treatments within each time point based on LSD tests.

**Table 2:** Abbott-corrected mortality of adult *Amrasca biguttula* across insecticides, doses, and time points. Values represent SD mortality (n = 3 dishes per treatment). Acute and residual periods represent distinct exposure cohorts, not repeated observations on the same insects. The 5 h and 24 h values correspond to the first cohort introduced immediately after treatment. The 72 h values correspond to a second cohort introduced at 24 h onto 24 h-old residues, and the 96 h values correspond to a third cohort introduced at 72 h onto 72 h-old residues. Thus, each time point reflects residue age at evaluation, rather than continuous tracking of a single cohort. The final row summarizes the overall statistical significance from GLM/ANOVA models at 5 h. Dose labels (D1–D5) correspond to the five concentrations tested for each insecticide: Tolfenpyrad = 0.80, 1.26, 2.00, 3.16, and 5.00 mL L^−1^; Flupyradifurone = 0.12, 0.21, 0.35, 0.59, and 1.00 mL L^−1^; Bifenthrin = 0.20, 0.30, 0.45, 0.67, and 1.00 g L^−1^. An untreated control was included at each dose position (T4D1–T4D5).

Treatment	Dose	5	24	72	96
Bifenthrin (Talstar)	D1	20.0 ± 20.0 a	86.7 ± 11.5 a	100.0 ± 0.0 a	40.0 ± 0.0 a
	D2	20.0 ± 20.0 a	86.7 ± 11.5 a	100.0 ± 0.0 a	40.0 ± 0.0 a
	D3	33.3 ± 11.5 a	100.0 ± 0.0 a	100.0 ± 0.0 a	40.0 ± 0.0 a
	D4	53.3 ± 30.6 a	100.0 ± 0.0 a	100.0 ± 0.0 a	40.0 ± 0.0 a
	D5	66.7 ± 41.6 a	100.0 ± 0.0 a	100.0 ± 0.0 a	40.0 ± 0.0 a
Control	D1	0.0 ± 0.0 b	20.0 ± 34.6 b	0.0 ± 0.0 b	0.0 ± 0.0 b
	D2	0.0 ± 0.0 b	6.7 ± 11.5 b	0.0 ± 0.0 b	0.0 ± 0.0 b
	D3	0.0 ± 0.0 b	53.3 ± 50.3 b	0.0 ± 0.0 b	0.0 ± 0.0 b
	D4	26.7 ± 46.2 b	0.0 ± 0.0 b	0.0 ± 0.0 b	0.0 ± 0.0 b
	D5	13.3 ± 23.1 b	20.0 ± 20.0 b	0.0 ± 0.0 b	0.0 ± 0.0 b
Flupyradifurone (Altus)	D1	13.3 ± 11.5 a	66.7 ± 23.1 a	100.0 ± 0.0 a	40.0 ± 0.0 a
	D2	20.0 ± 20.0 a	86.7 ± 11.5 a	100.0 ± 0.0 a	40.0 ± 0.0 a
	D3	26.7 ± 30.6 a	60.0 ± 52.9 a	100.0 ± 0.0 a	40.0 ± 0.0 a
	D4	26.7 ± 23.1 a	66.7 ± 57.7 a	100.0 ± 0.0 a	40.0 ± 0.0 a
	D5	80.0 ± 34.6 a	100.0 ± 0.0 a	100.0 ± 0.0 a	40.0 ± 0.0 a
Tolfenpyrad (Hachi Hachi)	D1	20.0 ± 34.6 a	60.0 ± 52.9 a	100.0 ± 0.0 a	40.0 ± 0.0 a
	D2	20.0 ± 20.0 a	100.0 ± 0.0 a	100.0 ± 0.0 a	40.0 ± 0.0 a
	D3	20.0 ± 20.0 a	86.7 ± 11.5 a	100.0 ± 0.0 a	40.0 ± 0.0 a
	D4	20.0 ± 34.6 a	53.3 ± 50.3 a	100.0 ± 0.0 a	40.0 ± 0.0 a
	D5	93.3 ± 11.5 a	100.0 ± 0.0 a	100.0 ± 0.0 a	40.0 ± 0.0 a
Statistical analysis (Treatment effect)		*F* = 4.57, *p* = 0.0076	*F* = 19.18, *p* < 0.0001	*F* = 2.66 × 10^31^, *p* < 0.0001	*F* = 1.63 × 10^30^, *p* < 0.0001

Note: Letters indicate mean separation among insecticide treatments within each time point based on LSD tests.

**Table 3:** Minimum effective dose (MED) and time to 90% mortality (T90) for *Amrasca biguttula* by insecticide and life stage. MED is defined as the lowest tested dose achieving ≥ 90% mean mortality. T90 is the earliest time point at which ≥90% mean mortality was observed. These descriptive potency metrics were used in place of LC_50_ values because mortality reached ceiling levels across most treatments.

Life Stage	Treatment	MED	T90
Adult	Bifenthrin (Talstar)	—	24
Adult	Control	—	—
Adult	Flupyradifurone (Altus)	—	72
Adult	Tolfenpyrad (Hachi Hachi)	—	72
Immature	Bifenthrin (Talstar)	D1	24
Immature	Control	—	—
Immature	Flupyradifurone (Altus)	D1	24
Immature	Tolfenpyrad (Hachi Hachi)	D3	24

Note: The em dash (—) indicates cases in which ≥90% mortality occurred across all doses or time points, preventing identification of a minimum effective dose (MED) or the earliest time to 90% mortality (T90).

## Data Availability

The original contributions presented in this study are included in the article/Supplementary Materials. Further inquiries can be directed to the corresponding author.

## Supplementary Materials

The following supporting information can be downloaded at https://www.mdpi.com/article/10.3390/insects17040358/s1: Supplementary Figure S1. Preparation and setup of treated hibiscus leaves used in the leaf-disc bioassay. Newly fully expanded hibiscus leaves were rinsed with distilled water, air-dried, and dipped into insecticide solutions until fully immersed. After blotting excess solution, treated leaves were placed into 9 cm plastic Petri dishes fitted with ventilated lids containing a 3 cm no-thrips mesh screen. Leaves were allowed to air-dry for 45 min before insects were introduced. Adult and immature *A. biguttula* were transferred using a fine camel-hair brush to minimize handling stress and natural mortality. The figure shows the randomized arrangement of Petri dishes inside the environmental chamber during the assay; Supplementary Figure S2. Corrected mortality of *Amrasca biguttula* adults (A) and immatures (B) across insecticides, dose levels, and post-treatment intervals. Panels show mean (±SE) corrected mortality for adults (A) and immature stages (B) exposed to five dose levels (D1–D5) of each insecticide at 5, 24, 72, and 96 h after treatment. Each point represents the mean of three replicate dishes (five insects per dish), with lines connecting dose-specific trajectories within each insecticide. The 5–24 h interval represents the acute exposure phase, while the 72–96 h interval reflects residual effects measured on a separate cohort; Supplementary Figure S3. Dose-indexed corrected mortality at 24 h for adult *Amrasca biguttula*. Corrected mortality at 24 h is plotted against the log_10_-transformed dose index (D1–D5) for each insecticide. Points represent mean (±SE) mortality across three replicate dishes, and fitted lines represent linear trends included for visualization only. Adult mortality approached or reached ceiling levels for most insecticides at 24 h, resulting in minimal dose-dependent variation. Because the dataset lacked a graded mortality response, these plots serve as descriptive summaries rather than inferential dose–response models. The absence of partial mortality across doses confirms that LC_50_ estimation was not appropriate for this dataset; Supplementary Figure S4. Dose-indexed corrected mortality at 24 h for immature *Amrasca biguttula*. Mean (±SE) corrected mortality at 24 h is shown for immatures across all doses and insecticides. Immature mortality reached 100% for all insecticides at all doses by 24 h, producing flat, ceiling-level profiles. Fitted lines are included for visualization but do not represent inferential dose–response models. These results further support the use of descriptive potency metrics (MED and T90) rather than LC_50_ estimation, as the dataset lacked the partial mortality range required for probit or log-logistic modeling; Supplementary Table S1. Raw mortality data for *Amrasca biguttula* adults and immatures across all insecticides, dose levels, exposure periods, and replicates. Values represent the number of dead insects out of five per dish. These raw data were used to compute corrected mortality, fit binomial GLM and ANOVA models, calculate Minimum Effective Dose (MED) and T90 values, and generate Kaplan–Meier survival curves.
