# Supplementary material for: First Evaluation of Insecticide Efficacy Against the Invasive Two-Spot Cotton Leafhopper (Amrasca biguttula [Hemiptera: Cicadellidae]) on Ornamental Hibiscus in the United States
Source: Insects. 2026 Mar 25;17(4):358. doi: 10.3390/insects17040358 (PMC13115638; doi:10.3390/insects17040358)
Supplement: Supplementary file 1 [file insects-17-00358-s001.zip › Supplementary Table S1.pdf]

Supplementary Table S1. Raw mortality data for *Amrasca biguttula* across all insecticides, doses, time points, and replicates. Values represent the number of dead insects out of five per dish. These data were used to compute corrected mortality, GLM/ANOVA models, and Kaplan–Meier survival curves.

Life Stage	HAT	Treatment	Dose	Replicate	Dead	Total	Mortality
Adult	5	Tolfenpyrad (Hachi Hachi)	D1	1	0	5	0
Adult	5	Tolfenpyrad (Hachi Hachi)	D1	2	0	5	0
Adult	5	Tolfenpyrad (Hachi Hachi)	D1	3	3	5	60
Adult	24	Tolfenpyrad (Hachi Hachi)	D1	1	0	5	0
Adult	24	Tolfenpyrad (Hachi Hachi)	D1	2	5	5	100
Adult	24	Tolfenpyrad (Hachi Hachi)	D1	3	4	5	80
Adult	72	Tolfenpyrad (Hachi Hachi)	D1	1	5	5	100
Adult	72	Tolfenpyrad (Hachi Hachi)	D1	2	5	5	100
Adult	72	Tolfenpyrad (Hachi Hachi)	D1	3	5	5	100
Adult	96	Tolfenpyrad (Hachi Hachi)	D1	1	2	5	40
Adult	96	Tolfenpyrad (Hachi Hachi)	D1	2	2	5	40
Adult	96	Tolfenpyrad (Hachi Hachi)	D1	3	2	5	40
Immature	5	Tolfenpyrad (Hachi Hachi)	D1	1	3	5	60
Immature	5	Tolfenpyrad (Hachi Hachi)	D1	2	1	5	20
Immature	5	Tolfenpyrad (Hachi Hachi)	D1	3	1	5	20
Immature	24	Tolfenpyrad (Hachi Hachi)	D1	1	5	5	100
Immature	24	Tolfenpyrad (Hachi Hachi)	D1	2	5	5	100
Immature	24	Tolfenpyrad (Hachi Hachi)	D1	3	5	5	100
Immature	72	Tolfenpyrad (Hachi Hachi)	D1	1	5	5	100
Immature	72	Tolfenpyrad (Hachi Hachi)	D1	2	5	5	100
Immature	72	Tolfenpyrad (Hachi Hachi)	D1	3	5	5	100
Immature	96	Tolfenpyrad (Hachi Hachi)	D1	1	5	5	100
Immature	96	Tolfenpyrad (Hachi Hachi)	D1	2	5	5	100
Immature	96	Tolfenpyrad (Hachi Hachi)	D1	3	5	5	100
Adult	5	Flupyradifurone (Altus)	D1	1	1	5	20
Adult	5	Flupyradifurone (Altus)	D1	2	1	5	20
Adult	5	Flupyradifurone (Altus)	D1	3	0	5	0
Adult	24	Flupyradifurone (Altus)	D1	1	2	5	40
Adult	24	Flupyradifurone (Altus)	D1	2	4	5	80
Adult	24	Flupyradifurone (Altus)	D1	3	4	5	80
Adult	72	Flupyradifurone (Altus)	D1	1	5	5	100
Adult	72	Flupyradifurone (Altus)	D1	2	5	5	100
Adult	72	Flupyradifurone (Altus)	D1	3	5	5	100
Adult	96	Flupyradifurone (Altus)	D1	1	2	5	40
Adult	96	Flupyradifurone (Altus)	D1	2	2	5	40
Adult	96	Flupyradifurone (Altus)	D1	3	2	5	40
Immature	5	Flupyradifurone (Altus)	D1	1	2	5	40
Immature	5	Flupyradifurone (Altus)	D1	2	2	5	40
Immature	5	Flupyradifurone (Altus)	D1	3	5	5	100
Immature	24	Flupyradifurone (Altus)	D1	1	5	5	100
Immature	24	Flupyradifurone (Altus)	D1	2	5	5	100

Life Stage	HAT	Treatment	Dose	Replicate	Dead	Total	Mortality
Immature	24	Flupyradifurone (Altus)	D1	3	5	5	100
Immature	72	Flupyradifurone (Altus)	D1	1	5	5	100
Immature	72	Flupyradifurone (Altus)	D1	2	5	5	100
Immature	72	Flupyradifurone (Altus)	D1	3	5	5	100
Immature	96	Flupyradifurone (Altus)	D1	1	5	5	100
Immature	96	Flupyradifurone (Altus)	D1	2	5	5	100
Immature	96	Flupyradifurone (Altus)	D1	3	5	5	100
Adult	5	Bifenthrin (Talstar)	D1	1	2	5	40
Adult	5	Bifenthrin (Talstar)	D1	2	0	5	0
Adult	5	Bifenthrin (Talstar)	D1	3	1	5	20
Adult	24	Bifenthrin (Talstar)	D1	1	4	5	80
Adult	24	Bifenthrin (Talstar)	D1	2	4	5	80
Adult	24	Bifenthrin (Talstar)	D1	3	5	5	100
Adult	72	Bifenthrin (Talstar)	D1	1	5	5	100
Adult	72	Bifenthrin (Talstar)	D1	2	5	5	100
Adult	72	Bifenthrin (Talstar)	D1	3	5	5	100
Adult	96	Bifenthrin (Talstar)	D1	1	2	5	40
Adult	96	Bifenthrin (Talstar)	D1	2	2	5	40
Adult	96	Bifenthrin (Talstar)	D1	3	2	5	40
Immature	5	Bifenthrin (Talstar)	D1	1	2	5	40
Immature	5	Bifenthrin (Talstar)	D1	2	3	5	60
Immature	5	Bifenthrin (Talstar)	D1	3	4	5	80
Immature	24	Bifenthrin (Talstar)	D1	1	5	5	100
Immature	24	Bifenthrin (Talstar)	D1	2	5	5	100
Immature	24	Bifenthrin (Talstar)	D1	3	5	5	100
Immature	72	Bifenthrin (Talstar)	D1	1	5	5	100
Immature	72	Bifenthrin (Talstar)	D1	2	5	5	100
Immature	72	Bifenthrin (Talstar)	D1	3	5	5	100
Immature	96	Bifenthrin (Talstar)	D1	1	5	5	100
Immature	96	Bifenthrin (Talstar)	D1	2	5	5	100
Immature	96	Bifenthrin (Talstar)	D1	3	5	5	100
Adult	5	Control	D1	1	0	5	0
Adult	5	Control	D1	2	0	5	0
Adult	5	Control	D1	3	0	5	0
Adult	24	Control	D1	1	0	5	0
Adult	24	Control	D1	2	0	5	0
Adult	24	Control	D1	3	3	5	60
Adult	72	Control	D1	1	0	5	0
Adult	72	Control	D1	2	0	5	0
Adult	72	Control	D1	3	0	5	0
Adult	96	Control	D1	1	0	5	0
Adult	96	Control	D1	2	0	5	0
Adult	96	Control	D1	3	0	5	0
Immature	5	Control	D1	1	0	5	0
Immature	5	Control	D1	2	0	5	0

Life Stage	HAT	Treatment	Dose	Replicate	Dead	Total	Mortality
Immature	5	Control	D1	3	0	5	0
Immature	24	Control	D1	1	0	5	0
Immature	24	Control	D1	2	0	5	0
Immature	24	Control	D1	3	0	5	0
Immature	72	Control	D1	1	0	5	0
Immature	72	Control	D1	2	0	5	0
Immature	72	Control	D1	3	0	5	0
Immature	96	Control	D1	1	0	5	0
Immature	96	Control	D1	2	0	5	0
Immature	96	Control	D1	3	0	5	0
Adult	5	Tolfenpyrad (Hachi Hachi)	D2	1	1	5	20
Adult	5	Tolfenpyrad (Hachi Hachi)	D2	2	0	5	0
Adult	5	Tolfenpyrad (Hachi Hachi)	D2	3	2	5	40
Adult	24	Tolfenpyrad (Hachi Hachi)	D2	1	5	5	100
Adult	24	Tolfenpyrad (Hachi Hachi)	D2	2	5	5	100
Adult	24	Tolfenpyrad (Hachi Hachi)	D2	3	5	5	100
Adult	72	Tolfenpyrad (Hachi Hachi)	D2	1	5	5	100
Adult	72	Tolfenpyrad (Hachi Hachi)	D2	2	5	5	100
Adult	72	Tolfenpyrad (Hachi Hachi)	D2	3	5	5	100
Adult	96	Tolfenpyrad (Hachi Hachi)	D2	1	2	5	40
Adult	96	Tolfenpyrad (Hachi Hachi)	D2	2	2	5	40
Adult	96	Tolfenpyrad (Hachi Hachi)	D2	3	2	5	40
Immature	5	Tolfenpyrad (Hachi Hachi)	D2	1	1	5	20
Immature	5	Tolfenpyrad (Hachi Hachi)	D2	2	4	5	80
Immature	5	Tolfenpyrad (Hachi Hachi)	D2	3	2	5	40
Immature	24	Tolfenpyrad (Hachi Hachi)	D2	1	5	5	100
Immature	24	Tolfenpyrad (Hachi Hachi)	D2	2	5	5	100
Immature	24	Tolfenpyrad (Hachi Hachi)	D2	3	5	5	100
Immature	72	Tolfenpyrad (Hachi Hachi)	D2	1	5	5	100
Immature	72	Tolfenpyrad (Hachi Hachi)	D2	2	5	5	100
Immature	72	Tolfenpyrad (Hachi Hachi)	D2	3	5	5	100
Immature	96	Tolfenpyrad (Hachi Hachi)	D2	1	5	5	100
Immature	96	Tolfenpyrad (Hachi Hachi)	D2	2	5	5	100
Immature	96	Tolfenpyrad (Hachi Hachi)	D2	3	5	5	100
Adult	5	Flupyradifurone (Altus)	D2	1	0	5	0
Adult	5	Flupyradifurone (Altus)	D2	2	1	5	20
Adult	5	Flupyradifurone (Altus)	D2	3	2	5	40
Adult	24	Flupyradifurone (Altus)	D2	1	4	5	80
Adult	24	Flupyradifurone (Altus)	D2	2	4	5	80
Adult	24	Flupyradifurone (Altus)	D2	3	5	5	100
Adult	72	Flupyradifurone (Altus)	D2	1	5	5	100
Adult	72	Flupyradifurone (Altus)	D2	2	5	5	100
Adult	72	Flupyradifurone (Altus)	D2	3	5	5	100
Adult	96	Flupyradifurone (Altus)	D2	1	2	5	40
Adult	96	Flupyradifurone (Altus)	D2	2	2	5	40

Life Stage	HAT	Treatment	Dose	Replicate	Dead	Total	Mortality
Adult	96	Flupyradifurone (Altus)	D2	3	2	5	40
Immature	5	Flupyradifurone (Altus)	D2	1	4	5	80
Immature	5	Flupyradifurone (Altus)	D2	2	1	5	20
Immature	5	Flupyradifurone (Altus)	D2	3	1	5	20
Immature	24	Flupyradifurone (Altus)	D2	1	5	5	100
Immature	24	Flupyradifurone (Altus)	D2	2	5	5	100
Immature	24	Flupyradifurone (Altus)	D2	3	5	5	100
Immature	72	Flupyradifurone (Altus)	D2	1	5	5	100
Immature	72	Flupyradifurone (Altus)	D2	2	5	5	100
Immature	72	Flupyradifurone (Altus)	D2	3	5	5	100
Immature	96	Flupyradifurone (Altus)	D2	1	5	5	100
Immature	96	Flupyradifurone (Altus)	D2	2	5	5	100
Immature	96	Flupyradifurone (Altus)	D2	3	5	5	100
Adult	5	Bifenthrin (Talstar)	D2	1	0	5	0
Adult	5	Bifenthrin (Talstar)	D2	2	2	5	40
Adult	5	Bifenthrin (Talstar)	D2	3	1	5	20
Adult	24	Bifenthrin (Talstar)	D2	1	4	5	80
Adult	24	Bifenthrin (Talstar)	D2	2	5	5	100
Adult	24	Bifenthrin (Talstar)	D2	3	4	5	80
Adult	72	Bifenthrin (Talstar)	D2	1	5	5	100
Adult	72	Bifenthrin (Talstar)	D2	2	5	5	100
Adult	72	Bifenthrin (Talstar)	D2	3	5	5	100
Adult	96	Bifenthrin (Talstar)	D2	1	2	5	40
Adult	96	Bifenthrin (Talstar)	D2	2	2	5	40
Adult	96	Bifenthrin (Talstar)	D2	3	2	5	40
Immature	5	Bifenthrin (Talstar)	D2	1	4	5	80
Immature	5	Bifenthrin (Talstar)	D2	2	4	5	80
Immature	5	Bifenthrin (Talstar)	D2	3	3	5	60
Immature	24	Bifenthrin (Talstar)	D2	1	5	5	100
Immature	24	Bifenthrin (Talstar)	D2	2	5	5	100
Immature	24	Bifenthrin (Talstar)	D2	3	5	5	100
Immature	72	Bifenthrin (Talstar)	D2	1	5	5	100
Immature	72	Bifenthrin (Talstar)	D2	2	5	5	100
Immature	72	Bifenthrin (Talstar)	D2	3	5	5	100
Immature	96	Bifenthrin (Talstar)	D2	1	5	5	100
Immature	96	Bifenthrin (Talstar)	D2	2	5	5	100
Immature	96	Bifenthrin (Talstar)	D2	3	5	5	100
Adult	5	Control	D2	1	0	5	0
Adult	5	Control	D2	2	0	5	0
Adult	5	Control	D2	3	0	5	0
Adult	24	Control	D2	1	0	5	0
Adult	24	Control	D2	2	0	5	0
Adult	24	Control	D2	3	1	5	20
Adult	72	Control	D2	1	0	5	0
Adult	72	Control	D2	2	0	5	0

Life Stage	HAT	Treatment	Dose	Replicate	Dead	Total	Mortality
Adult	72	Control	D2	3	0	5	0
Adult	96	Control	D2	1	0	5	0
Adult	96	Control	D2	2	0	5	0
Adult	96	Control	D2	3	0	5	0
Immature	5	Control	D2	1	1	5	20
Immature	5	Control	D2	2	0	5	0
Immature	5	Control	D2	3	0	5	0
Immature	24	Control	D2	1	0	5	0
Immature	24	Control	D2	2	0	5	0
Immature	24	Control	D2	3	0	5	0
Immature	72	Control	D2	1	0	5	0
Immature	72	Control	D2	2	0	5	0
Immature	72	Control	D2	3	0	5	0
Immature	96	Control	D2	1	0	5	0
Immature	96	Control	D2	2	0	5	0
Immature	96	Control	D2	3	5	5	100
Adult	5	Tolfenpyrad (Hachi Hachi)	D3	1	1	5	20
Adult	5	Tolfenpyrad (Hachi Hachi)	D3	2	2	5	40
Adult	5	Tolfenpyrad (Hachi Hachi)	D3	3	0	5	0
Adult	24	Tolfenpyrad (Hachi Hachi)	D3	1	5	5	100
Adult	24	Tolfenpyrad (Hachi Hachi)	D3	2	4	5	80
Adult	24	Tolfenpyrad (Hachi Hachi)	D3	3	4	5	80
Adult	72	Tolfenpyrad (Hachi Hachi)	D3	1	5	5	100
Adult	72	Tolfenpyrad (Hachi Hachi)	D3	2	5	5	100
Adult	72	Tolfenpyrad (Hachi Hachi)	D3	3	5	5	100
Adult	96	Tolfenpyrad (Hachi Hachi)	D3	1	2	5	40
Adult	96	Tolfenpyrad (Hachi Hachi)	D3	2	2	5	40
Adult	96	Tolfenpyrad (Hachi Hachi)	D3	3	2	5	40
Immature	5	Tolfenpyrad (Hachi Hachi)	D3	1	4	5	80
Immature	5	Tolfenpyrad (Hachi Hachi)	D3	2	5	5	100
Immature	5	Tolfenpyrad (Hachi Hachi)	D3	3	4	5	80
Immature	24	Tolfenpyrad (Hachi Hachi)	D3	1	5	5	100
Immature	24	Tolfenpyrad (Hachi Hachi)	D3	2	5	5	100
Immature	24	Tolfenpyrad (Hachi Hachi)	D3	3	5	5	100
Immature	72	Tolfenpyrad (Hachi Hachi)	D3	1	5	5	100
Immature	72	Tolfenpyrad (Hachi Hachi)	D3	2	5	5	100
Immature	72	Tolfenpyrad (Hachi Hachi)	D3	3	5	5	100
Immature	96	Tolfenpyrad (Hachi Hachi)	D3	1	5	5	100
Immature	96	Tolfenpyrad (Hachi Hachi)	D3	2	5	5	100
Immature	96	Tolfenpyrad (Hachi Hachi)	D3	3	5	5	100
Adult	5	Flupyradifurone (Altus)	D3	1	0	5	0
Adult	5	Flupyradifurone (Altus)	D3	2	1	5	20
Adult	5	Flupyradifurone (Altus)	D3	3	3	5	60
Adult	24	Flupyradifurone (Altus)	D3	1	0	5	0
Adult	24	Flupyradifurone (Altus)	D3	2	4	5	80

Life Stage	HAT	Treatment	Dose	Replicate	Dead	Total	Mortality
Adult	24	Flupyradifurone (Altus)	D3	3	5	5	100
Adult	72	Flupyradifurone (Altus)	D3	1	5	5	100
Adult	72	Flupyradifurone (Altus)	D3	2	5	5	100
Adult	72	Flupyradifurone (Altus)	D3	3	5	5	100
Adult	96	Flupyradifurone (Altus)	D3	1	2	5	40
Adult	96	Flupyradifurone (Altus)	D3	2	2	5	40
Adult	96	Flupyradifurone (Altus)	D3	3	2	5	40
Immature	5	Flupyradifurone (Altus)	D3	1	5	5	100
Immature	5	Flupyradifurone (Altus)	D3	2	2	5	40
Immature	5	Flupyradifurone (Altus)	D3	3	3	5	60
Immature	24	Flupyradifurone (Altus)	D3	1	5	5	100
Immature	24	Flupyradifurone (Altus)	D3	2	5	5	100
Immature	24	Flupyradifurone (Altus)	D3	3	5	5	100
Immature	72	Flupyradifurone (Altus)	D3	1	5	5	100
Immature	72	Flupyradifurone (Altus)	D3	2	5	5	100
Immature	72	Flupyradifurone (Altus)	D3	3	5	5	100
Immature	96	Flupyradifurone (Altus)	D3	1	5	5	100
Immature	96	Flupyradifurone (Altus)	D3	2	5	5	100
Immature	96	Flupyradifurone (Altus)	D3	3	5	5	100
Adult	5	Bifenthrin (Talstar)	D3	1	2	5	40
Adult	5	Bifenthrin (Talstar)	D3	2	1	5	20
Adult	5	Bifenthrin (Talstar)	D3	3	2	5	40
Adult	24	Bifenthrin (Talstar)	D3	1	5	5	100
Adult	24	Bifenthrin (Talstar)	D3	2	5	5	100
Adult	24	Bifenthrin (Talstar)	D3	3	5	5	100
Adult	72	Bifenthrin (Talstar)	D3	1	5	5	100
Adult	72	Bifenthrin (Talstar)	D3	2	5	5	100
Adult	72	Bifenthrin (Talstar)	D3	3	5	5	100
Adult	96	Bifenthrin (Talstar)	D3	1	2	5	40
Adult	96	Bifenthrin (Talstar)	D3	2	2	5	40
Adult	96	Bifenthrin (Talstar)	D3	3	2	5	40
Immature	5	Bifenthrin (Talstar)	D3	1	5	5	100
Immature	5	Bifenthrin (Talstar)	D3	2	3	5	60
Immature	5	Bifenthrin (Talstar)	D3	3	3	5	60
Immature	24	Bifenthrin (Talstar)	D3	1	5	5	100
Immature	24	Bifenthrin (Talstar)	D3	2	5	5	100
Immature	24	Bifenthrin (Talstar)	D3	3	5	5	100
Immature	72	Bifenthrin (Talstar)	D3	1	5	5	100
Immature	72	Bifenthrin (Talstar)	D3	2	5	5	100
Immature	72	Bifenthrin (Talstar)	D3	3	5	5	100
Immature	96	Bifenthrin (Talstar)	D3	1	5	5	100
Immature	96	Bifenthrin (Talstar)	D3	2	5	5	100
Immature	96	Bifenthrin (Talstar)	D3	3	5	5	100
Adult	5	Control	D3	1	0	5	0
Adult	5	Control	D3	2	0	5	0

Life Stage	HAT	Treatment	Dose	Replicate	Dead	Total	Mortality
Adult	5	Control	D3	3	0	5	0
Adult	24	Control	D3	1	0	5	0
Adult	24	Control	D3	2	3	5	60
Adult	24	Control	D3	3	5	5	100
Adult	72	Control	D3	1	0	5	0
Adult	72	Control	D3	2	0	5	0
Adult	72	Control	D3	3	0	5	0
Adult	96	Control	D3	1	0	5	0
Adult	96	Control	D3	2	0	5	0
Adult	96	Control	D3	3	0	5	0
Immature	5	Control	D3	1	4	5	80
Immature	5	Control	D3	2	0	5	0
Immature	5	Control	D3	3	0	5	0
Immature	24	Control	D3	1	0	5	0
Immature	24	Control	D3	2	0	5	0
Immature	24	Control	D3	3	0	5	0
Immature	72	Control	D3	1	0	5	0
Immature	72	Control	D3	2	0	5	0
Immature	72	Control	D3	3	0	5	0
Immature	96	Control	D3	1	0	5	0
Immature	96	Control	D3	2	0	5	0
Immature	96	Control	D3	3	0	5	0
Adult	5	Tolfenpyrad (Hachi Hachi)	D4	1	0	5	0
Adult	5	Tolfenpyrad (Hachi Hachi)	D4	2	3	5	60
Adult	5	Tolfenpyrad (Hachi Hachi)	D4	3	0	5	0
Adult	24	Tolfenpyrad (Hachi Hachi)	D4	1	5	5	100
Adult	24	Tolfenpyrad (Hachi Hachi)	D4	2	3	5	60
Adult	24	Tolfenpyrad (Hachi Hachi)	D4	3	0	5	0
Adult	72	Tolfenpyrad (Hachi Hachi)	D4	1	5	5	100
Adult	72	Tolfenpyrad (Hachi Hachi)	D4	2	5	5	100
Adult	72	Tolfenpyrad (Hachi Hachi)	D4	3	5	5	100
Adult	96	Tolfenpyrad (Hachi Hachi)	D4	1	2	5	40
Adult	96	Tolfenpyrad (Hachi Hachi)	D4	2	2	5	40
Adult	96	Tolfenpyrad (Hachi Hachi)	D4	3	2	5	40
Immature	5	Tolfenpyrad (Hachi Hachi)	D4	1	4	5	80
Immature	5	Tolfenpyrad (Hachi Hachi)	D4	2	2	5	40
Immature	5	Tolfenpyrad (Hachi Hachi)	D4	3	4	5	80
Immature	24	Tolfenpyrad (Hachi Hachi)	D4	1	5	5	100
Immature	24	Tolfenpyrad (Hachi Hachi)	D4	2	5	5	100
Immature	24	Tolfenpyrad (Hachi Hachi)	D4	3	5	5	100
Immature	72	Tolfenpyrad (Hachi Hachi)	D4	1	5	5	100
Immature	72	Tolfenpyrad (Hachi Hachi)	D4	2	5	5	100
Immature	72	Tolfenpyrad (Hachi Hachi)	D4	3	5	5	100
Immature	96	Tolfenpyrad (Hachi Hachi)	D4	1	5	5	100
Immature	96	Tolfenpyrad (Hachi Hachi)	D4	2	5	5	100

Life Stage	HAT	Treatment	Dose	Replicate	Dead	Total	Mortality
Immature	96	Tolfenpyrad (Hachi Hachi)	D4	3	5	5	100
Adult	5	Flupyradifurone (Altus)	D4	1	2	5	40
Adult	5	Flupyradifurone (Altus)	D4	2	0	5	0
Adult	5	Flupyradifurone (Altus)	D4	3	2	5	40
Adult	24	Flupyradifurone (Altus)	D4	1	5	5	100
Adult	24	Flupyradifurone (Altus)	D4	2	0	5	0
Adult	24	Flupyradifurone (Altus)	D4	3	5	5	100
Adult	72	Flupyradifurone (Altus)	D4	1	5	5	100
Adult	72	Flupyradifurone (Altus)	D4	2	5	5	100
Adult	72	Flupyradifurone (Altus)	D4	3	5	5	100
Adult	96	Flupyradifurone (Altus)	D4	1	2	5	40
Adult	96	Flupyradifurone (Altus)	D4	2	2	5	40
Adult	96	Flupyradifurone (Altus)	D4	3	2	5	40
Immature	5	Flupyradifurone (Altus)	D4	1	5	5	100
Immature	5	Flupyradifurone (Altus)	D4	2	5	5	100
Immature	5	Flupyradifurone (Altus)	D4	3	4	5	80
Immature	24	Flupyradifurone (Altus)	D4	1	5	5	100
Immature	24	Flupyradifurone (Altus)	D4	2	5	5	100
Immature	24	Flupyradifurone (Altus)	D4	3	5	5	100
Immature	72	Flupyradifurone (Altus)	D4	1	5	5	100
Immature	72	Flupyradifurone (Altus)	D4	2	5	5	100
Immature	72	Flupyradifurone (Altus)	D4	3	5	5	100
Immature	96	Flupyradifurone (Altus)	D4	1	5	5	100
Immature	96	Flupyradifurone (Altus)	D4	2	5	5	100
Immature	96	Flupyradifurone (Altus)	D4	3	5	5	100
Adult	5	Bifenthrin (Talstar)	D4	1	4	5	80
Adult	5	Bifenthrin (Talstar)	D4	2	1	5	20
Adult	5	Bifenthrin (Talstar)	D4	3	3	5	60
Adult	24	Bifenthrin (Talstar)	D4	1	5	5	100
Adult	24	Bifenthrin (Talstar)	D4	2	5	5	100
Adult	24	Bifenthrin (Talstar)	D4	3	5	5	100
Adult	72	Bifenthrin (Talstar)	D4	1	5	5	100
Adult	72	Bifenthrin (Talstar)	D4	2	5	5	100
Adult	72	Bifenthrin (Talstar)	D4	3	5	5	100
Adult	96	Bifenthrin (Talstar)	D4	1	2	5	40
Adult	96	Bifenthrin (Talstar)	D4	2	2	5	40
Adult	96	Bifenthrin (Talstar)	D4	3	2	5	40
Immature	5	Bifenthrin (Talstar)	D4	1	5	5	100
Immature	5	Bifenthrin (Talstar)	D4	2	4	5	80
Immature	5	Bifenthrin (Talstar)	D4	3	5	5	100
Immature	24	Bifenthrin (Talstar)	D4	1	5	5	100
Immature	24	Bifenthrin (Talstar)	D4	2	5	5	100
Immature	24	Bifenthrin (Talstar)	D4	3	5	5	100
Immature	72	Bifenthrin (Talstar)	D4	1	5	5	100
Immature	72	Bifenthrin (Talstar)	D4	2	5	5	100

Life Stage	HAT	Treatment	Dose	Replicate	Dead	Total	Mortality
Immature	72	Bifenthrin (Talstar)	D4	3	5	5	100
Immature	96	Bifenthrin (Talstar)	D4	1	5	5	100
Immature	96	Bifenthrin (Talstar)	D4	2	5	5	100
Immature	96	Bifenthrin (Talstar)	D4	3	5	5	100
Adult	5	Control	D4	1	4	5	80
Adult	5	Control	D4	2	0	5	0
Adult	5	Control	D4	3	0	5	0
Adult	24	Control	D4	1	0	5	0
Adult	24	Control	D4	2	0	5	0
Adult	24	Control	D4	3	0	5	0
Adult	72	Control	D4	1	0	5	0
Adult	72	Control	D4	2	0	5	0
Adult	72	Control	D4	3	0	5	0
Adult	96	Control	D4	1	0	5	0
Adult	96	Control	D4	2	0	5	0
Adult	96	Control	D4	3	0	5	0
Immature	5	Control	D4	1	0	5	0
Immature	5	Control	D4	2	0	5	0
Immature	5	Control	D4	3	0	5	0
Immature	24	Control	D4	1	0	5	0
Immature	24	Control	D4	2	0	5	0
Immature	24	Control	D4	3	0	5	0
Immature	72	Control	D4	1	0	5	0
Immature	72	Control	D4	2	0	5	0
Immature	72	Control	D4	3	0	5	0
Immature	96	Control	D4	1	0	5	0
Immature	96	Control	D4	2	0	5	0
Immature	96	Control	D4	3	0	5	0
Adult	5	Tolfenpyrad (Hachi Hachi)	D5	1	5	5	100
Adult	5	Tolfenpyrad (Hachi Hachi)	D5	2	5	5	100
Adult	5	Tolfenpyrad (Hachi Hachi)	D5	3	4	5	80
Adult	24	Tolfenpyrad (Hachi Hachi)	D5	1	5	5	100
Adult	24	Tolfenpyrad (Hachi Hachi)	D5	2	5	5	100
Adult	24	Tolfenpyrad (Hachi Hachi)	D5	3	5	5	100
Adult	72	Tolfenpyrad (Hachi Hachi)	D5	1	5	5	100
Adult	72	Tolfenpyrad (Hachi Hachi)	D5	2	5	5	100
Adult	72	Tolfenpyrad (Hachi Hachi)	D5	3	5	5	100
Adult	96	Tolfenpyrad (Hachi Hachi)	D5	1	2	5	40
Adult	96	Tolfenpyrad (Hachi Hachi)	D5	2	2	5	40
Adult	96	Tolfenpyrad (Hachi Hachi)	D5	3	2	5	40
Immature	5	Tolfenpyrad (Hachi Hachi)	D5	1	4	5	80
Immature	5	Tolfenpyrad (Hachi Hachi)	D5	2	5	5	100
Immature	5	Tolfenpyrad (Hachi Hachi)	D5	3	4	5	80
Immature	24	Tolfenpyrad (Hachi Hachi)	D5	1	5	5	100
Immature	24	Tolfenpyrad (Hachi Hachi)	D5	2	5	5	100

Life Stage	HAT	Treatment	Dose	Replicate	Dead	Total	Mortality
Immature	24	Tolfenpyrad (Hachi Hachi)	D5	3	5	5	100
Immature	72	Tolfenpyrad (Hachi Hachi)	D5	1	5	5	100
Immature	72	Tolfenpyrad (Hachi Hachi)	D5	2	5	5	100
Immature	72	Tolfenpyrad (Hachi Hachi)	D5	3	5	5	100
Immature	96	Tolfenpyrad (Hachi Hachi)	D5	1	5	5	100
Immature	96	Tolfenpyrad (Hachi Hachi)	D5	2	5	5	100
Immature	96	Tolfenpyrad (Hachi Hachi)	D5	3	5	5	100
Adult	5	Flupyradifurone (Altus)	D5	1	2	5	40
Adult	5	Flupyradifurone (Altus)	D5	2	5	5	100
Adult	5	Flupyradifurone (Altus)	D5	3	5	5	100
Adult	24	Flupyradifurone (Altus)	D5	1	5	5	100
Adult	24	Flupyradifurone (Altus)	D5	2	5	5	100
Adult	24	Flupyradifurone (Altus)	D5	3	5	5	100
Adult	72	Flupyradifurone (Altus)	D5	1	5	5	100
Adult	72	Flupyradifurone (Altus)	D5	2	5	5	100
Adult	72	Flupyradifurone (Altus)	D5	3	5	5	100
Adult	96	Flupyradifurone (Altus)	D5	1	2	5	40
Adult	96	Flupyradifurone (Altus)	D5	2	2	5	40
Adult	96	Flupyradifurone (Altus)	D5	3	2	5	40
Immature	5	Flupyradifurone (Altus)	D5	1	5	5	100
Immature	5	Flupyradifurone (Altus)	D5	2	4	5	80
Immature	5	Flupyradifurone (Altus)	D5	3	5	5	100
Immature	24	Flupyradifurone (Altus)	D5	1	5	5	100
Immature	24	Flupyradifurone (Altus)	D5	2	5	5	100
Immature	24	Flupyradifurone (Altus)	D5	3	5	5	100
Immature	72	Flupyradifurone (Altus)	D5	1	5	5	100
Immature	72	Flupyradifurone (Altus)	D5	2	5	5	100
Immature	72	Flupyradifurone (Altus)	D5	3	5	5	100
Immature	96	Flupyradifurone (Altus)	D5	1	5	5	100
Immature	96	Flupyradifurone (Altus)	D5	2	5	5	100
Immature	96	Flupyradifurone (Altus)	D5	3	5	5	100
Adult	5	Bifenthrin (Talstar)	D5	1	4	5	80
Adult	5	Bifenthrin (Talstar)	D5	2	1	5	20
Adult	5	Bifenthrin (Talstar)	D5	3	5	5	100
Adult	24	Bifenthrin (Talstar)	D5	1	5	5	100
Adult	24	Bifenthrin (Talstar)	D5	2	5	5	100
Adult	24	Bifenthrin (Talstar)	D5	3	5	5	100
Adult	72	Bifenthrin (Talstar)	D5	1	5	5	100
Adult	72	Bifenthrin (Talstar)	D5	2	5	5	100
Adult	72	Bifenthrin (Talstar)	D5	3	5	5	100
Adult	96	Bifenthrin (Talstar)	D5	1	2	5	40
Adult	96	Bifenthrin (Talstar)	D5	2	2	5	40
Adult	96	Bifenthrin (Talstar)	D5	3	2	5	40
Immature	5	Bifenthrin (Talstar)	D5	1	3	5	60
Immature	5	Bifenthrin (Talstar)	D5	2	5	5	100

Life Stage	HAT	Treatment	Dose	Replicate	Dead	Total	Mortality
Immature	5	Bifenthrin (Talstar)	D5	3	5	5	100
Immature	24	Bifenthrin (Talstar)	D5	1	5	5	100
Immature	24	Bifenthrin (Talstar)	D5	2	5	5	100
Immature	24	Bifenthrin (Talstar)	D5	3	5	5	100
Immature	72	Bifenthrin (Talstar)	D5	1	5	5	100
Immature	72	Bifenthrin (Talstar)	D5	2	5	5	100
Immature	72	Bifenthrin (Talstar)	D5	3	5	5	100
Immature	96	Bifenthrin (Talstar)	D5	1	5	5	100
Immature	96	Bifenthrin (Talstar)	D5	2	5	5	100
Immature	96	Bifenthrin (Talstar)	D5	3	5	5	100
Adult	5	Control	D5	1	0	5	0
Adult	5	Control	D5	2	2	5	40
Adult	5	Control	D5	3	0	5	0
Adult	24	Control	D5	1	0	5	0
Adult	24	Control	D5	2	2	5	40
Adult	24	Control	D5	3	1	5	20
Adult	72	Control	D5	1	0	5	0
Adult	72	Control	D5	2	0	5	0
Adult	72	Control	D5	3	0	5	0
Adult	96	Control	D5	1	0	5	0
Adult	96	Control	D5	2	0	5	0
Adult	96	Control	D5	3	0	5	0
Immature	5	Control	D5	1	0	5	0
Immature	5	Control	D5	2	0	5	0
Immature	5	Control	D5	3	5	5	100
Immature	24	Control	D5	1	0	5	0
Immature	24	Control	D5	2	0	5	0
Immature	24	Control	D5	3	5	5	100
Immature	72	Control	D5	1	0	5	0
Immature	72	Control	D5	2	0	5	0
Immature	72	Control	D5	3	0	5	0
Immature	96	Control	D5	1	0	5	0
Immature	96	Control	D5	2	0	5	0
Immature	96	Control	D5	3	0	5	0
