# Supplementary figures and images for: First Evaluation of Insecticide Efficacy Against the Invasive Two-Spot Cotton Leafhopper (Amrasca biguttula [Hemiptera: Cicadellidae]) on Ornamental Hibiscus in the United States
Source: Insects. 2026 Mar 25;17(4):358. doi: 10.3390/insects17040358 (PMC13115638; doi:10.3390/insects17040358)

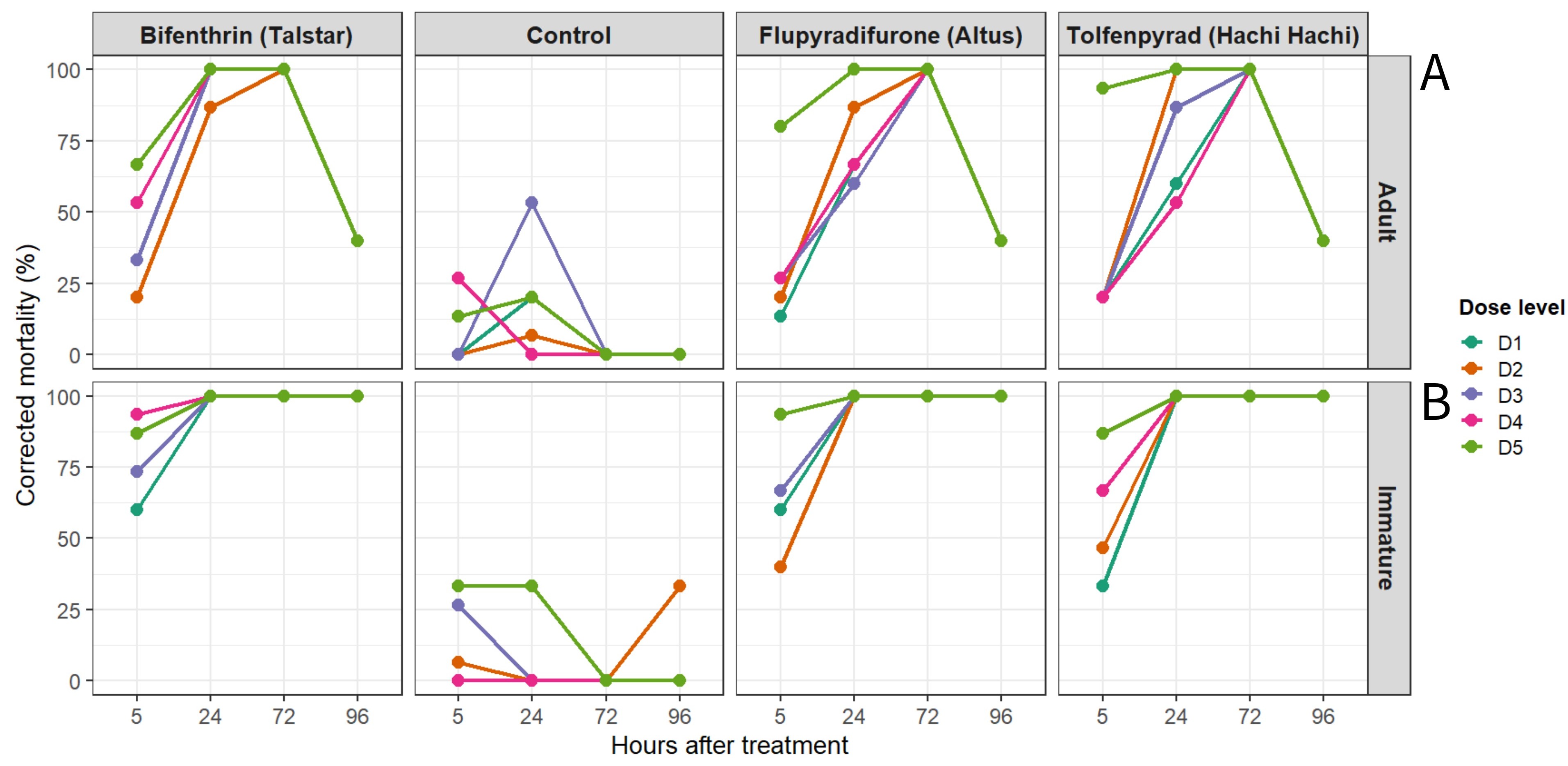

Supplement: Supplementary file 1 [file insects-17-00358-s001.zip › Supplementary Figure S2_updated.jpg]

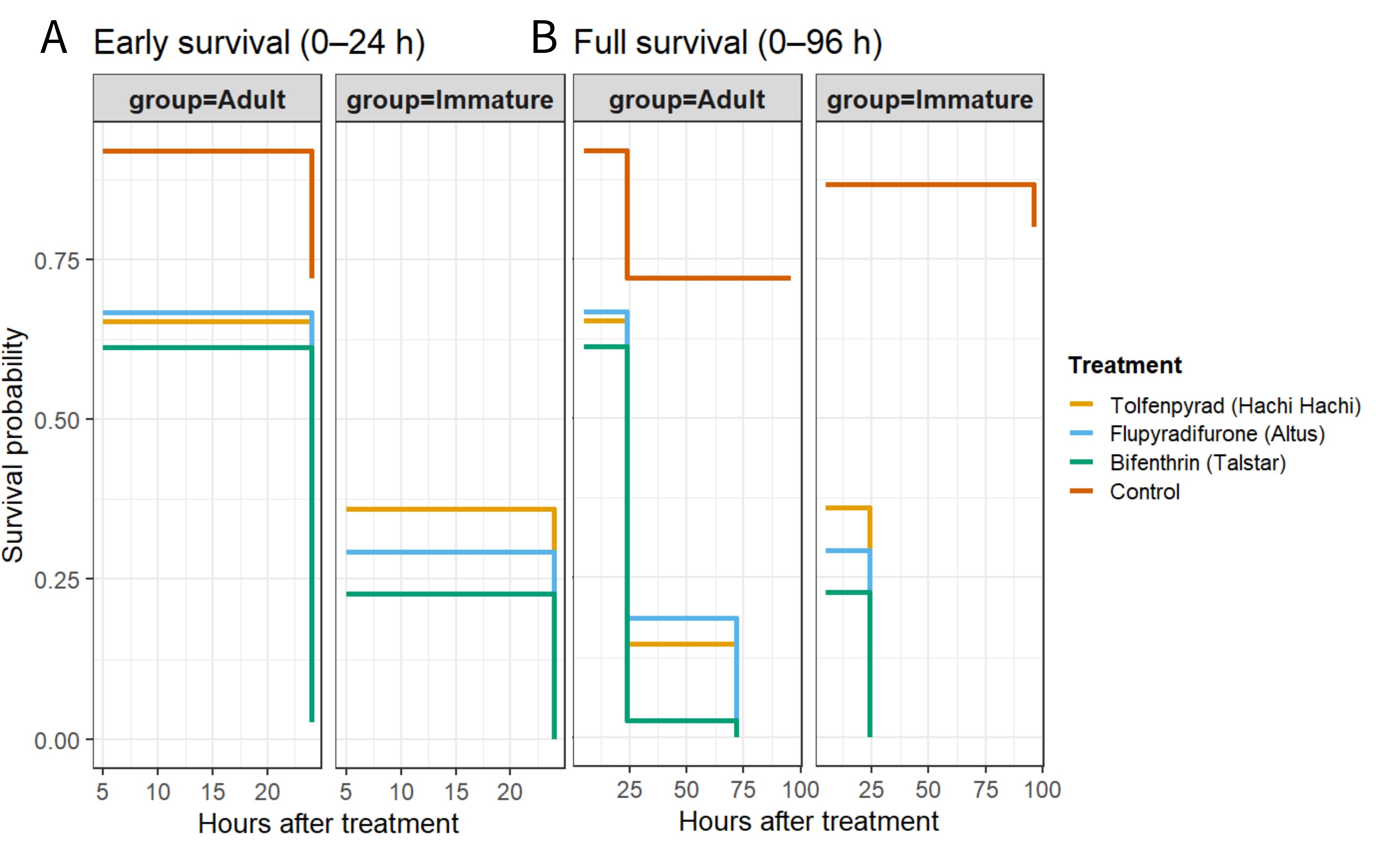

Supplement: Supplementary file 1 [file insects-17-00358-s001.zip › Supplementary Figure S3_updated.jpg]
